# Supplementary material for: A Euclidean transformer for fast and stable machine learned force fields
Source: Nat Commun. 2024 Aug 6;15:6539. doi: 10.1038/s41467-024-50620-6 (PMC11303804; doi:10.1038/s41467-024-50620-6)
Supplement: Supplementary file 1 — Supplementary Information [file 41467_2024_50620_MOESM1_ESM.pdf]

Supplementary Information

A Euclidean Transformer for Fast and Stable Machine Learned Force Fields

Appendix A: SI Experiments

1. MD Simulations

In Fig. 1 we plot the distribution of total energy values, energies as a function of time and the temperature as a function of time for the MD simulations which have been used to calculate the power spectra (main body Fig. 5). As it can be readily verified, the performed simulations are energy conserving where the total energies follow a Gaussian distribution with a small, but non-zero variance that is a consequence of the finite step size in the numerical integration performed in the Velocity-Verlet update.

Fig. 2 (b) shows the power spectra for Ac-Ala3-NHMe at three different temperatures of 100 K, 300 K and 500 K. As reported for DHA in the main body of the text, we find non-trivial shifts in frequency and population across different temperatures. On the right hand side, we show the radial and angular distribution functions for both Ac-Ala3-NHMe and DHA at a temperature of 500 K.

2. Radial Distribution Functions

From the MD simulations for the small organic molecules from the MD17 data set, we further calculated radial distribution functions (RDFs) and compare them to the RDFs from DFT. The results are displayed in Fig. 3.

3. DHA Data Efficiency

To measure the data efficiency of DHA, we trained models with different l_{max} for varying N_{train} and do a linear fit in the log-log space.

4. Invariant vs. Equivariant

In Tab. 1 we report the parameters found by fitting a log-normal curve to the error distributions. As written in the main body of the text, we used two different error metrics. The per-atom force MSE is calculated as

$$d_i = \sqrt{\sum_{\alpha \in (x,y,z)} (F_{i,\alpha} - F_{i,\alpha}^{\text{GT}})^2} \tag{A1}$$

and the per-structure MSE is computed as

$$D_k = \frac{1}{n} \sum_{i=1}^{n} d_i, \tag{A2}$$

involving an additional mean per structure. From the equations above its clear that the mean for both d_i and D_k is identical, whereas the variances can be differ.

5. Minima Hopping

a. *Stable Minima*

During the minima hopping algorithm the LBFGS optimization for DHA (Ac-Ala3-NHMe) did not converge in 8 (15) cases. Since they are comparably large in energy they are rejected due to E_{diff} and consequently do not affect the algorithm during runtime. When comparing all minima that have been visited, however, we have to explicitly exclude them. We do this by first choosing the minima with the lowest potential energy as reference structure and

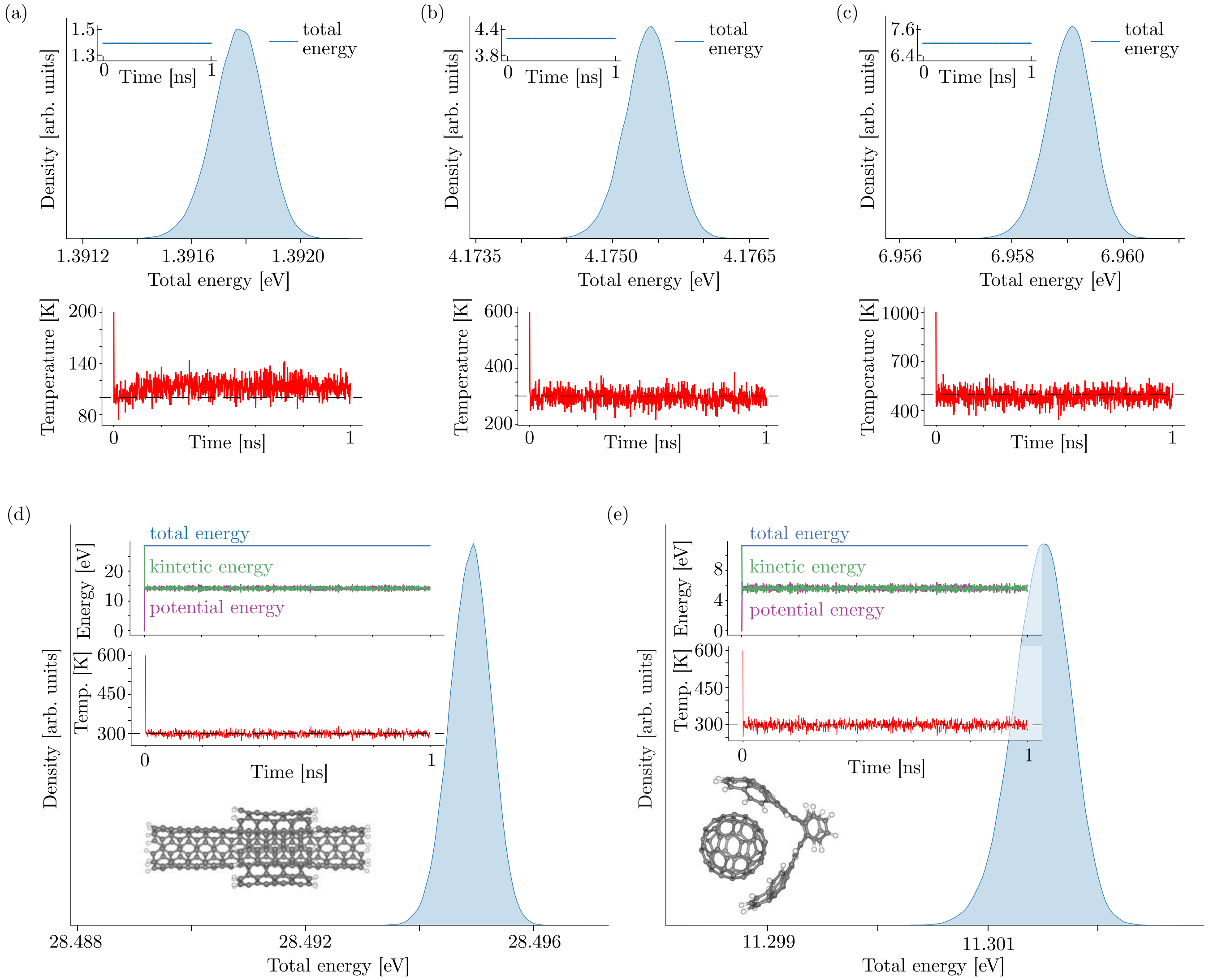

Supplementary Figure 1. **Molecular dynamics statistics.** (a, b, c) Total energy distribution, total energy over time (inset) and the temperature over time as observed in the MD simulations for DHA with target temperatures 100 K (a), 300 K (b) and 500 K (c) using the Velocity-Verlet algorithm. From the resulting trajectories, the power spectra reported in the main body of the text have been calculated. (d, e) Total energy distribution as well as total energy, potential energy, kinetic energy and temperature as a function of time (insets) for the double walled nanotube (d) and the buckyball catcher (e). After a few ps, kinetic and potential energy reach equilibration leading to the desired target temperature of 300 K.

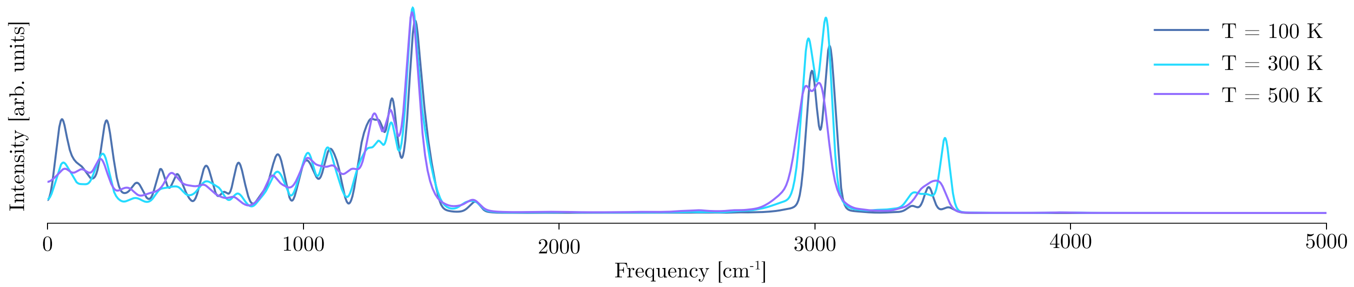

Supplementary Figure 2. **Temperature dependence of power spectra**. Power spectra for Ac-Ala3-NHMe at different temperatures obtained with SO3krates.

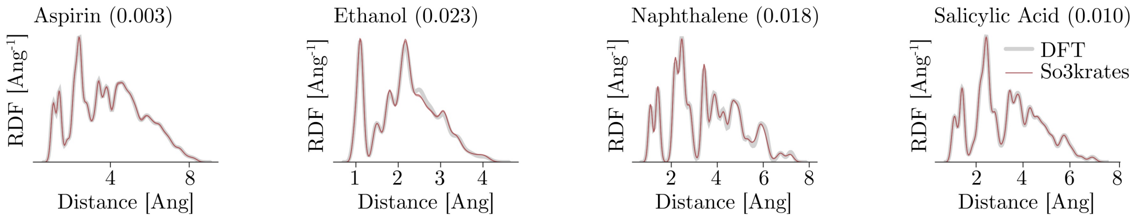

Supplementary Figure 3. **Observables on MD17**. Radial distribution functions (RDFs) obtained from the MD simulations for which stabilities and FPS have been reported in the main part of the manuscript. For each structure the RDF for each of the five runs is plotted, which shows that observables are stable over multiple runs and structures. The number in brackets corresponds to the MAE between the RDFs obtained from SO3KRATES and from DFT.

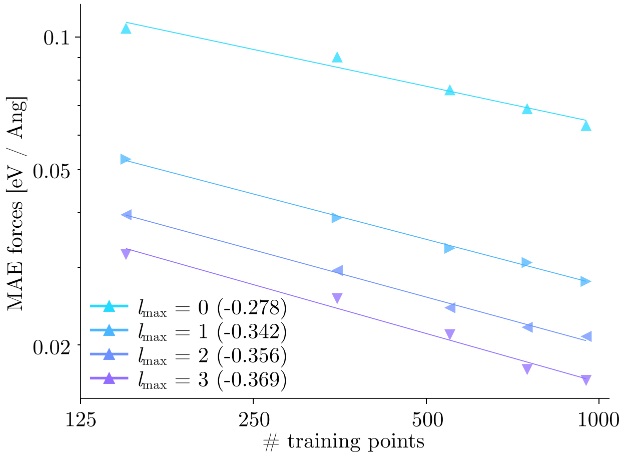

Supplementary Figure 4. **Data efficiency.** Figure shows the data efficiency measured in terms of force approximation error for the DHA molecule for different maximal degree l_{max} in the SO3KRATES network. With increasing l_{max}, we find increasing data efficiency, which is calculated as the slope in the log-log plot. The largest difference for both, accuracy and data efficiency can be found when going from an invariant ($l_{\mathrm{max}} = 0$) to an equivariant model ($l_{\mathrm{max}} > 0$).

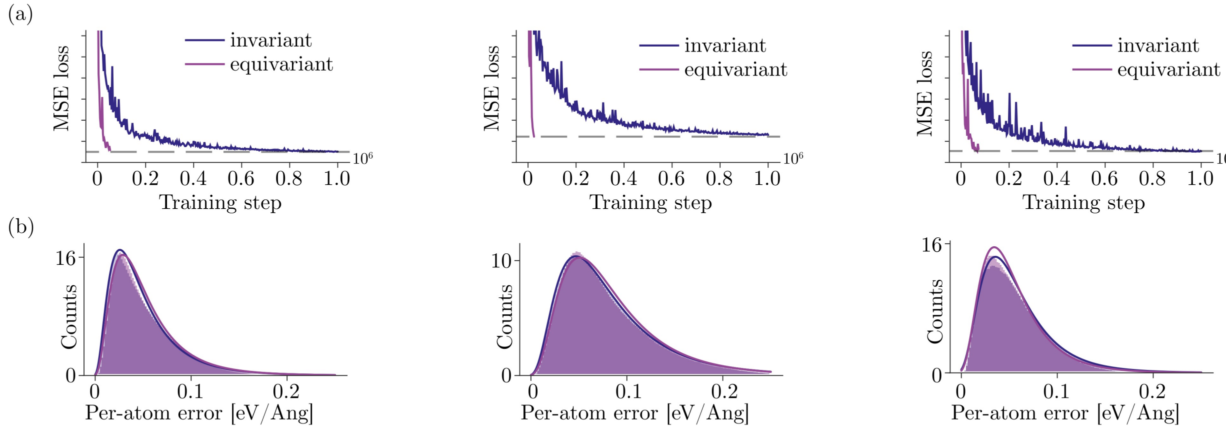

Supplementary Figure 5. **Training curves and error distribution**. Plots from left to the right correspond to the structures Ac-Ala3-NHMe, DHA and the Adenine-Thymine pair (AT-AT). (a) Validation loss for an invariant ($l_{\mathrm{max}} = 0$) and an equivariant ($l_{\mathrm{max}} = 3$) SO3KRATES model observed during training, where the training of the equivariant model is stopped as soon as it reaches the error of the invariant model. (b) Per-atom error distributions for an invariant and an equivariant SO3KRATES model trained with two different model seeds. Spread and mean of the error distributions are given in Supplementary Tab. 1.

	per atom error d_i				per structure error D_k			
	$l_{\max} = 0$		$l_{\max} = 3$		$l_{\max} = 0$		$l_{\max} = 3$	
Ac-Ala3-NHMe	$\mu = 0.051$	$s = 0.641$	$\mu = 0.053$	$s = 0.610$	$\mu = 0.051$	$s = 0.269$	$\mu = 0.053$	$s = 0.169$
DHA	$\mu = 0.083$	$s = 0.591$	$\mu = 0.083$	$s = 0.554$	$\mu = 0.083$	$s = 0.198$	$\mu = 0.083$	$s = 0.155$
AT-AT	$\mu = 0.057$	$s = 0.511$	$\mu = 0.055$	$s = 0.243$	$\mu = 0.057$	$s = 0.501$	$\mu = 0.055$	$s = 0.186$

Supplementary Table 1. **Error analysis**. Mean and spread of the per-atom MSE d_i (cf. Eq. (A1)) and of the per-structure MSE D_k (cf. Eq. (A2)) for the three different structures investigated in the main text.

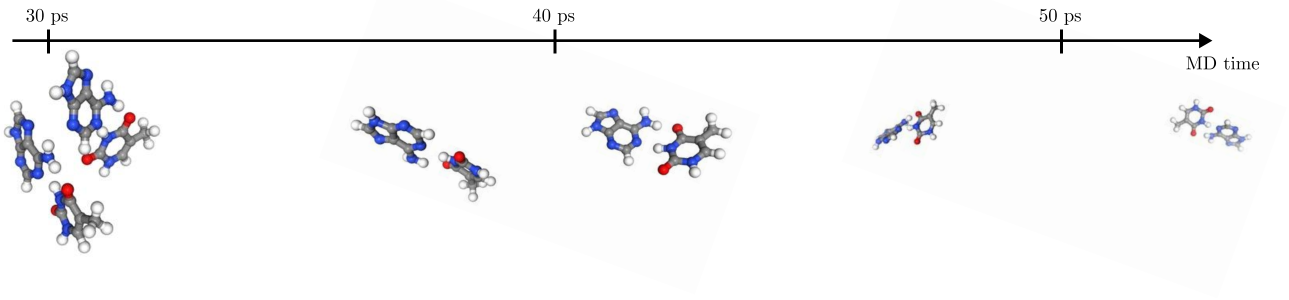

Supplementary Figure 6. **Breaking of pi-bonds during simulation of AT-AT.** Breaking of a π-π bond between AT-AT complexes over a time interval of 20 ps during a molecular dynamics simulation.

calculate the bond lengths from it. Afterwards, we compare the bond lengths of all other visited minima to this reference structure and exclude them when the RMSD between all bond lengths is larger than 10^{-2}. We note, that this allows to detect "bad" minima in a self-contained manner without the need of any re-calculations with *ab-initio* methods. Further, we re-calculated the minima with different optimizer settings and found the new minima to be stable. Thus, the failed optimizations were due to the hyperparameters of the optimizer and not due to the MLFF.

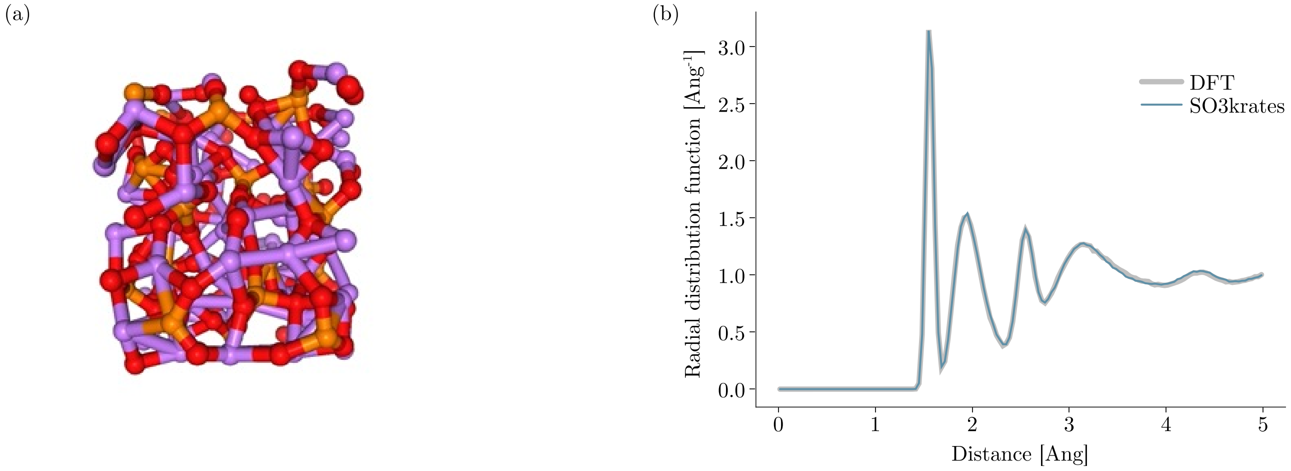

Supplementary Figure 7. **Application to materials**. (a) Li_3PO_4 structure in the quenched phase. The shown conformation corresponds to the starting conformation for the MD simulation with SO3KRATES. (b) Radial distribution function obtained from the last 20ps of a 50 ps MD simulation at 600 K, compared to the RDF from DFT.

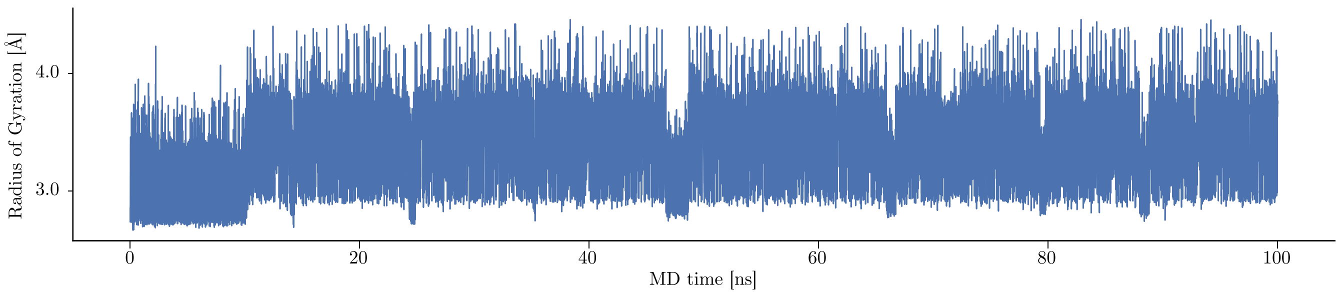

Supplementary Figure 8. **Radius of Gyration over time.** Radius of gyration as a function of simulation time for AcAla3NHMe.

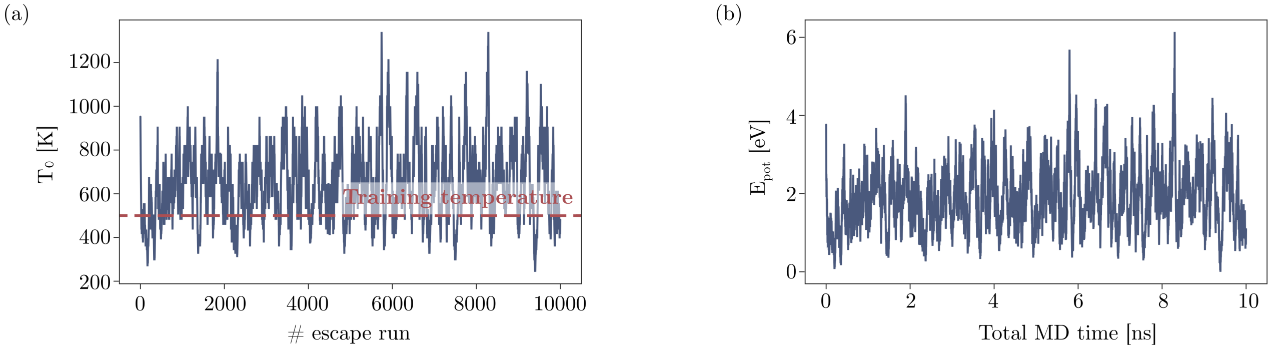

Supplementary Figure 9. **Minima hopping**. (a) The initial temperature T_0 for the MD simulations as a function of the MD escape run in the minima hopping algorithm. Since velocity-verlet is used for the MD simulation and the structure is in a local minima at the beginning of the MD, equipartition principle will result in an MD that has temperature $T_0/2$. (b) The maximal potential energy that is observed during each MD escape run vs the total MD simulation time.

b. Invariant Model

We additionally perform the minima hopping algorithm with an invariant SO3KRATES model. After a few escape runs, a dissociated minima is found as lowest minima. As a consequence, no new minima can be accepted in the following and the initial temperature starts to decrease towards zero (Fig. 10). The resulting minima lead to an non-physical representation of the PES, as it can be seen in Fig. 6 in the main body of the text. For the invariant model, we chose the one from the MD stability experiments, which has been found capable of producing partially stable MDs for Ac-Ala3-NHMe (Fig. 4).

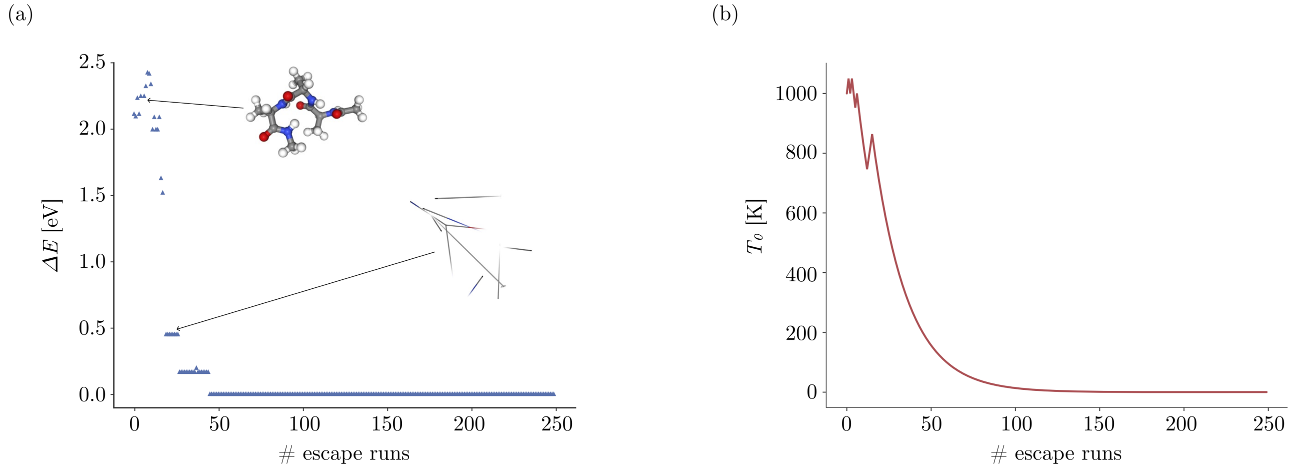

Supplementary Figure 10. **Minima hopping with an invariant model**. Figure shows the relative potential energy (a) as well as the initial temperature T_0 (b) observed during minima hopping with an invariant SO3krates model. After a few escape trials a dissociated structure is obtained as lowest energy minima. Since no lower minima is found, the initial temperature starts to decrease towards zero over escape runs.

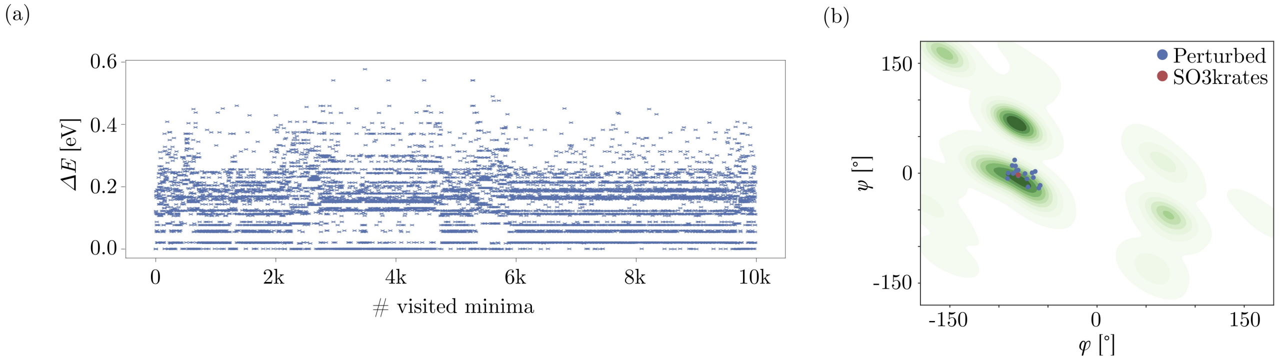

Supplementary Figure 11. **Minima hopping for other molecules**. (a) Potential energies for the visited minima of the Ac-Ala3-NHMe structure are shown. (b) Location in the Ramachandran plot of 18 randomly perturbed structures (blue) around the original minimum (red). The location of the re-performed relaxations is also shown in red. Since all optimizations relaxed into the same, original minimum one can only see a single red dot.

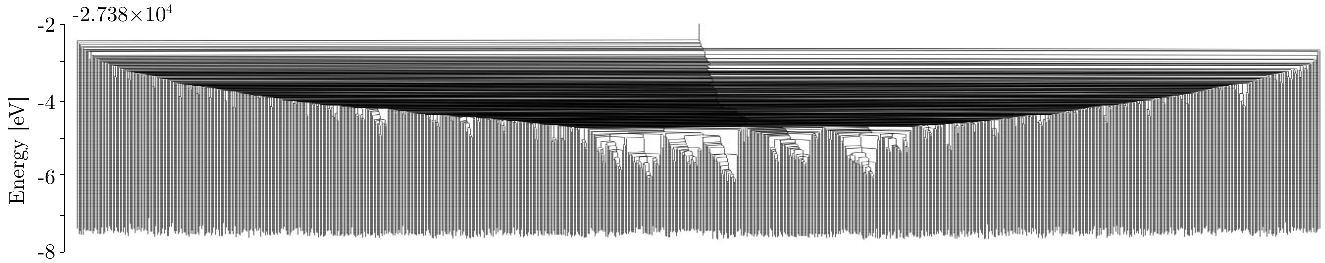

Supplementary Figure 12. **Disconnectivity graph for DHA.** One can construct so called disconnectivity graphs from the 10k minima of DHA which have been found using the minima hopping algorithm.
